# The temporoammonic input to the hippocampal CA1 region displays distinctly different synaptic plasticity compared to the Schaffer collateral input *in vivo*: significance for synaptic information processing

**DOI:** 10.3389/fnsyn.2013.00005

**Published:** 2013-08-23

**Authors:** Ayla Aksoy-Aksel, Denise Manahan-Vaughan

**Affiliations:** ^1^Department of Neurophysiology, Medical Faculty, Ruhr University BochumBochum, Germany; ^2^International Graduate School for Neuroscience, Ruhr University BochumBochum, Germany

**Keywords:** perforant path, synaptic plasticity, hippocampus, CA1, LTD, LTP, *in vivo*

## Abstract

In terms of its sub-regional differentiation, the hippocampal CA1 region receives cortical information directly via the perforant (temporoammonic) path (pp-CA1 synapse) and indirectly via the tri-synaptic pathway where the last relay station is the Schaffer collateral-CA1 synapse (Sc-CA1 synapse). Research to date on pp-CA1 synapses has been conducted predominantly *in vitro* and never in awake animals, but these studies hint that information processing at this synapse might be distinct to processing at the Sc-CA1 synapse. Here, we characterized synaptic properties and synaptic plasticity at the pp-CA1 synapse of freely behaving adult rats. We observed that field excitatory postsynaptic potentials at the pp-CA1 synapse have longer onset latencies and a shorter time-to-peak compared to the Sc-CA1 synapse. LTP (>24 h) was successfully evoked by tetanic afferent stimulation of pp-CA1 synapses. Low frequency stimulation evoked synaptic depression at Sc-CA1 synapses, but did not elicit LTD at pp-CA1 synapses unless the Schaffer collateral afferents to the CA1 region had been severed. Paired-pulse responses also showed significant differences. Our data suggest that synaptic plasticity at the pp-CA1 synapse is distinct from the Sc-CA1 synapse and that this may reflect its specific role in hippocampal information processing.

## Introduction

Accumulated knowledge derived from behavioral and electrophysiological research has enabled more detailed insights into the role of the different hippocampal subfields in learning and memory (Kemp and Manahan-Vaughan, 2004, 2008; Vago et al., 2007). Information to the hippocampal subfields is conveyed via the superficial layers II and III of entorhinal cortex (EC) via the perforant path. Layer II projects to the dentate gyrus (DG) and the CA3 regions, whereas layer III sends fibers mainly, if not exclusively, to the CA1 and the subiculum (Witter et al., 1988). In contrast to the trisynaptic input, which is diffusely widespread in terms of its connections (Ishizuka et al., 1990), the direct pathway from EC layer III to the CA1 is organized in a topographical way, forming almost a one-to-one connection. Moreover, the two inputs are separated anatomically with respect to the distance from the soma of the CA1 neurons. The basal dendrites as well as proximal part of the apical dendrites (CA1-stratum radiatum) are targets for the trisynaptic path whereas the distal part of apical dendrites (CA1-stratum lacunosum moleculare) is reserved for the perforant path synapses (Kajiwara et al., 2008). The two dendritic layers differ in terms of receptor composition (Nolan et al., 2004; Otmakhova et al., 2005; Ahmed and Siegelbaum, 2009) and molecular underpinnings of plasticity induction (Magee, 1999; Jarsky et al., 2005; Losonczy et al., 2008; Remy et al., 2009; Takahashi and Magee, 2009).

The functional role of the perforant (temporoammonic) path (pp)-CA1 synapse in information processing and storage in the hippocampal network is not yet clear, and this is largely due to the fact that electrophysiological studies of the synapse in freely behaving animals have not yet been carried out. Nevertheless, the role of the direct cortical input has been considered in many learning and memory models (Lisman and Otmakhova, 2001; Treves, 2004; Hasselmo and Eichenbaum, 2005; Rolls and Kesner, 2006). Since the CA1 comprises the only output from the hippocampus proper, it is proposed to comprise the final matching module (mismatch detector and information integrator) of information arising from both the CA3 and the direct sensory information from EC. This synapse may be important for the provision of an efference copy for information stored via the trisynaptic pathway (Lisman and Otmakhova, 2001) or for error detection (Izumi and Zorumski, 2008). Here, it may adopt a specific role in preliminary input differentiation according to the spatial and contextual characteristics of information (Ito and Schuman, 2012).

In order to begin to understand the role of synaptic transmission and plasticity at the pp-CA1 synapses in information storage and learning phenomena, we must examine this synapse in freely behaving animals. This was the objective of the present study. Having first established methods to enable the characterization of the pp-CA1 synapse, we studied the conditions under which synaptic plasticity is expressed and compared this to synaptic plasticity at the Sc-CA1 synapse. We established that the pp-CA1 synapse exhibits synaptic plasticity properties that differentiate it from all other hippocampal synapses studied to date in freely behaving rodents.

## Materials and methods

### Surgical procedures

The present study was carried out in accordance with the European Communities Council Directive of September 22nd, 2010 (2010/63/EU) for care of laboratory animals and after approval of the local ethics committee (Bezirksamt Arnsberg, Germany). All efforts were made to reduce the number of animals used.

Male Wistar rats (7–8 weeks old at the time of surgery) were implanted with electrodes (polyurethane-coated stainless steel wire; 1 mm in diameter) into the hippocampus and a cannula into the lateral cerebral ventricle. All surgical procedures were performed under anesthesia induced by intraperitoneal (i.p.) injection of sodium pentobarbital (52 mg/kg) the depth of anesthesia was regularly controlled via observing the EEG and by tail-pinch. If the animal showed signs of the approach of arousal a further injection was given. Body temperature was regulated by a heating blanket. The head was adjusted to have a negative slope of 1 mm between bregma and a fixed point 7 mm anterioposterior toward lambda (on the midline). The stereotaxic coordinates for the electrodes and the guiding cannula were referenced to the location of bregma in terms of anterioposterior (AP) and mediolateral (ML) distance. A cannula was inserted into the right lateral cerebral ventricle (0.5 mm posterior and 1.6 mm lateral to bregma with approximate depth of 4 mm).

**Figure 1:**
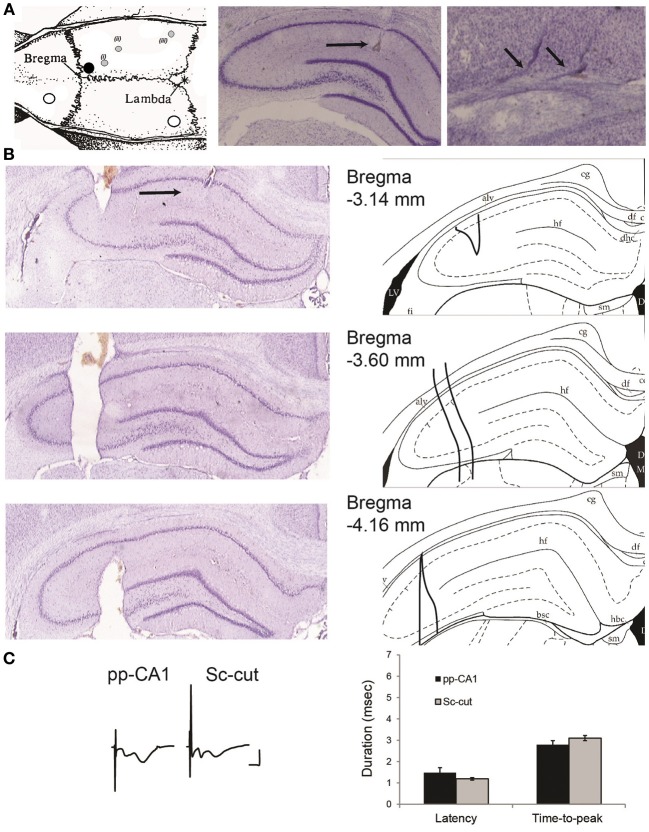
**(A)** Location of the drilled holes on the rat skull and electrodes. Left: the white circles indicate the position of the ground and reference screws. The black circle signifies the position of the guiding cannula that reaches the lateral ventricle. The gray circles show the planar position of (i) the recording electrode, (ii) the stimulating electrode for Schaffer collaterals and (iii) the stimulating electrode for the perforant path (angular bundle) [Modified from Paxinos and Watson (1998)]. The animals were implanted with a stimulation electrode either in the Schaffer collateral (Sc) fibers (ii) or angular bundle (iii) if not indicated otherwise. Middle: location of the recording electrode for the perforant path (pp)-CA1 synapse (black horizontal arrow). Right: tracks of a bipolar stimulating electrode at the angular bundle (small angled arrows). Middle and right: Nissl-stained hippocampal slices. **(B**) Histological verification of the severence of the Schaffer collateral input. Left: Nissl-stained hippocampal slices from an animal with severed Schaffer collateral input (black arrow points the recording site). Right: the corresponding drawings from the atlas for easier interpretation (Paxinos and Watson, 1998). Distance from bregma as indicated. alv, alveus of the hippocampus; df, dorsal fornix; hf, hippocampal fissure; LV, lateral ventricle. **(C)** fEPSP characteristics of intact and Sc-cut groups. Left: Examples of evoked potentials from intact (pp-CA1) and Sc severed (Sc-cut) animals. Vertical scale bar: 4 mV, horizontal scale bar: 5 ms. Right: Latency and time-to-peak values for the two groups (no significant difference, *n* = 6).

For fEPSP recordings in freely behaving rats, the animals were implanted unilaterally on the right hemisphere with a monopolar recording electrode and a bipolar stimulating electrode. For pp-CA1 and Sc-cut animals the recording electrode was positioned at the stratum lacunosum moleculare of the CA1 region (coordinates; from bregma, AP: −3.0; from midline, ML: +2.0 mm; from dura, DV: manually determined) and the stimulating electrode at the angular bundle with coordinates corresponding to the fibers of the medial perforant path (coordinates; from bregma, AP: −6.9; from midline, ML: +4.1 mm) (Figure 1A, middle and right picture, respectively). For Schaffer collateral-CA1 (Sc-CA1) animals the recording electrode was positioned at the stratum radiatum of the CA1 region (coordinates; from bregma, AP: −2.8; from midline, ML: +1.8 mm) and the stimulating electrode at the Schaffer collateral fibers (coordinates; from bregma, AP: −3.1; from midline, ML: +3.1 mm; from dura, DV: manually determined) (histology not shown) (Manahan-Vaughan and Reymann, 1995). Briefly, the dura was pierced and the electrodes were lowered slowly in the brain tissue. The evoked field excitatory postsynaptic potential (fEPSP) responses were monitored at different depths until a typical fEPSP was obtained. Except for the Sc-cut animals (described below) the electrodes were fixed with cyanoacrylate glue to the skull and the whole assembly was covered with dental cement. After surgery, the animals were placed under observation in a temperature-regulated environment. When they were fully awake and gave no signs of complications they were returned to their homecage and monitored closely for continued recovery. The wound was treated regularly with antibiotic powder. The animals were given the analgesic, meloxicam (0.2 mg/kg i.p.), to alleviate post-operative discomfort.

## Electrophysiological recordings

The *in vivo* experiments were carried out 7–10 days after the implantation of the electrodes. Basal synaptic transmission was recorded via test-pulse stimulation of the perforant path using a stimulation intensity determined from a previously established input/output (I/O) relationship (100–900 μ A) for each individual animal. The test-pulse intensity was determined as the intensity that elicits ca. 40% of the maximum fEPSP observed in the I/O curve. Stable responses could not be elicited from the pp-CA1 synapse using lower stimulus intensities. Data acquisition and storage was conducted with a personal computer (acquisition software: Intracell, Magdeburg). Responses were evoked by stimulating at frequency of 0.025 Hz with a single biphasic square wave pulse of 0.2 ms stimulus duration and 10 KHz sample rate (World Precision Instruments, USA), amplified via differential amplifier (A-M Systems, USA) and digitized (Cambridge Electronic Design, UK). For each time-point, five records of evoked responses were averaged. The cortical encephalogram (EEG) was monitored throughout the course of each experiment. The basal synaptic transmission was recorded every 5 min (average of 5 sweeps for every time point) for 75 min and every 15 min for the subsequent 225 min. In cases when synaptic plasticity was induced the stimulation protocol was applied after monitoring basal synaptic transmission (for at least 30 min) and continued for 4 h afterwards. The first 3 recordings after the stimulation were taken every 5 min and subsequently taken every 15 min.

Animals typically served as their own controls. This means, for example, that all plasticity effects were compared to the animals' own baseline response. Baseline recordings to evaluate stability of evoked responses over a 24 h period were conducted before plasticity experiments were commenced. If plasticity experiments were repeated in the same animal, an interval of minimally 7 days intervened. This was done to ensure that no residual plasticity effects influenced the new experiment. Test experiments were conducted to confirm that the sequence of plasticity experiments has no effect on experimental outcome (as long as ca. 7 days intervened between experiments). Lack of residual plasticity was confirmed by comparing the input-output curve on the nee experiment day with the input-output curve obtained prior to inducing plasticity ca. 7 days beforehand. Previous studies confirmed that LTP and LTD that endure for over 24 h and are elicited by similar afferent stimulation protocols, do not endure for longer than 1 week (Manahan-Vaughan and Braunewell, 1999; Kemp and Manahan-Vaughan, 2004).

The paired-pulse ratio was determined for five interpulse intervals (20, 25, 40, 50, and 100 ms) contiguously, separated by 40 s intervals. The procedure was repeated (20 min apart) maximum of five times for the low-intensity stimulation and three times for the high-intensity stimulation for every animal. Low-intensity corresponds to the stimulus intensity that elicits 40% of the maximal response obtained from the input-output relationship; high-intensity is double the low-intensity (or maximum of 400 μA).

For recordings in pp-CA1 high-frequency stimulation (HFS) protocols were employed. To induce short-term potentiation (STP) a single burst of 100 pulses at 100 Hz (0.1 ms stimulus duration) was applied. To induce long-term potentiation (LTP) (>4 h), four bursts of 100 pulses at 100 Hz (4 bursts of 100 stimuli, 0.1 ms stimulus duration, 10 s interburst interval) with an intertrain interval of 5 min were given, that has been reliably used to induce STP and LTP, respectively at the Sc-CA1 synapse (Kemp and Manahan-Vaughan, 2004). Additionally, a weak or strong tetanus of 200 Hz (3 bursts of 15 stimuli, 0.2 ms stimulus duration, 10 s interburst interval or 10 bursts of 15 stimuli, 0.2 ms stimulus duration, 10 s interburst interval) were applied to induce LTP and STP, respectively: these are protocols that are effective in inducing plasticity at the perforant path-dentate gyrus synapses *in vivo* (Kulla and Manahan-Vaughan, 2000, 2008; Manahan-Vaughan and Kulla, 2003).

In all hippocampal structures studied in our laboratory to date, low-frequency stimulation (LFS) consisting of 900 or 600 pulses at 1 Hz, has been shown to be effective in eliciting LTD and STD, respectively in Wistar rats (Manahan-Vaughan, 1997, 2000; Manahan-Vaughan and Braunewell, 1999; Kemp and Manahan-Vaughan, 2004; Poschel and Manahan-Vaughan, 2007; Hagena and Manahan-Vaughan, 2010). Therefore, this protocol was used to study synaptic depression at pp-CA1 synapses. During LFS the stimulus strength was either raised to 70% (i.e., the intensity that elicited an fEPSP that was 70% of the maximum seen in the I/O relationship) to elicit a more intense afferent stimulation, or was left at 40% level used for test-pulse stimulation.

The same protocols were employed for recordings in the Sc-CA1 synapse so as to enable comparison between the two inputs.

The slope of the evoked potentials was measured automatically via software controlled function as the maximum slope through the five steepest points from the beginning of the fEPSP and the first minimum located in the first deflection (Manahan-Vaughan, 1997).

### Severance of the schaffer collateral input

The Schaffer collateral input was severed in a group of animals to isolate the pp-CA1 synapse. After the final position of recording and stimulating electrodes was found, a stainless steel thin plate (with size of ca. 4.5 mm in height, 0.2–0.6 mm in width and 0.3–1.0 mm in length) was slowly lowered using the coordinates: 3.3 mm posterior to bregma and 3.3 mm lateral to the midline, until the whole length was inside the brain ipsilateral to the recording site. The tip was fixed with cyanoacrylate glue and the whole assembly was covered with dental cement. The extent of severance was examined histologically after the completion of *in vivo* experiments and found to cover approximately 1 mm of the septo-temporal axis (Figure 1B).

Severence of the Sc fibers did not affect the characteristics of fEPSPs in the CA1 region due to pp stimulation, although the pp-CA1 component became more, and the Sc-Ca1 component became less pronounced. The latency (delay between the stimulation artifact and the beginning of the potential) and time-to-peak (duration of the fEPSP from the beginning to the first minimum value) of the potentials remained unchanged: For six animals per group thirty fEPSPs were obtained in blocks of five (at 0.025 Hz) recorded at ca. 5 min intervals (30 min recording in total) and analyzed. The evoked responses revealed no significant difference between the Sc intact and Sc-cut conditions (*p* > 0.05, *n* = 6) (Figure 1C). Complex potentials were still evident following Schaffer collateral severance. This is presumably because not all of the Schaffer collaterals to the CA1 region were severed. What one sees, however, is that the CA1 component is much smaller and the pp-CA1 fEPSP is generally larger, in line with a predominant severance of Schaffer collaterals.

Theoretically it is possible to exclude the effect of volume conduction by spatially confined drug injections or local electrolytic lesions (both targeting the DG area directly below the recording site), but this strategy proved to be impractical for freely behaving conditions as the recording site was affected either by the spread of degeneration or because spared tissue at the target site still contaminated the evoked responses to an unpredictable extent (Aksoy-Aksel and Manahan-Vaughan, unpublished data). For this reason we did not employ chemical lesions of the DG (Leung et al., 1995) to examine the response within an “intact” hippocampal network.

### Histological analysis

The location of the recording and stimulation electrodes as well as the guiding cannula was verified by postmortem histological visualization. The tissue was fixed, coronal slices were obtained and Nissl stained (Manahan-Vaughan et al., 1998). Animals with misplaced electrodes were not included in the data analysis.

### Data analysis

For the analysis of the electrophysiological data, each time-point was calculated as the percentage of the average of the six recordings (pre-stimulation basal synaptic transmission values) for every subject individually. The graphs were constructed from the mean value of the percentages ± standard error of the mean (SEM). Between group statistics was done by two-way analysis of variance with repeated measures (ANOVA) by comparing the whole train of measurements after the (plasticity-inducing) stimulation. *Post-hoc* analysis with the Fisher's LSD test was used to test the significance of individual values in plasticity experiments, in cases where, for example the duration of an effect should be clarified. Student's *t*-test was used to assess the significance level for individual time points in paired-pulse and input-output curve experiments. *P* < 0.05 was considered significant.

## Results

### Establishment of electrophysiological procedures for recordings of pp-CA1 potentials in freely behaving rats

The electrophysiological evaluation of the pp-CA1 synapse has not been previously attempted by other researchers using freely behaving animals. As the methodological and research approach is therefore entirely novel, we first established procedures for electrophysiological recordings at the pp-CA1 synapse.

**Figure 2:**
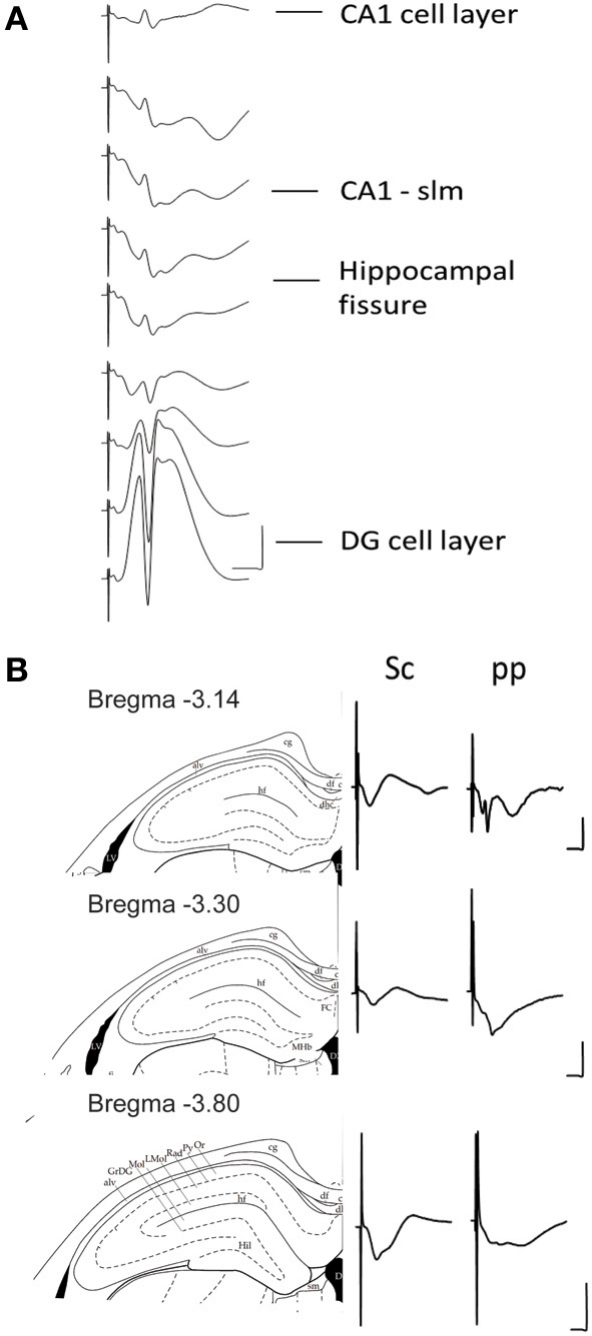
**(A)** Depth profile of evoked potentials as a result of perforant path stimulation. Example of typical field responses *in vivo* evoked by perforant path stimulation. The recordings started at the granule cell layer of the dentate gyrus (DG). The depth was decreased stepwise at 5 min intervals progressing “backwards” to the CA1 region. The distance between each representative recording was 160 μm. The stimulation intensity for all recordings was 200 μ A. Vertical scale bar: 5 mV, horizontal scale bar: 3 ms. **(B)** Evoked potentials at the CA1-stratum lacunosum moleculare synapse in response to Schaffer collateral and perforant path stimulation. Examples of typically evoked responses *in vivo* at the CA1-slm as a result of Schaffer collateral (analogs on the left) and perforant path stimulation (analogs traces on the right) at three different anterioposterior coordinates. The diagrams to the left of the analog traces represent coronal sections of hippocampal formation −3.14 mm (Top), −3.30 mm (middle), and −3.80 mm (bottom) from bregma (Paxinos and Watson, 1998). The histological examination of the recording site was depicted as a dot on the corresponding slide (Paxinos and Watson, 1998). All of the recordings were taken from freely behaving rats. The stimulation intensity for all recordings was 200 μ A. Vertical scale bar: 5 mV, horizontal scale bar: 5 ms for all traces. alv, alveus of the hippocampus; cg, cingulum; df, dorsal fornix; FC, fasciola cinereun; GrDG, granular layer of the dentate gyrus; hf, hippocampal fissure; Hil, hilus of the dentate gyrus; LMol, lacunosum moleculare layer of the hippocampus; Mol, molecular layer of the dentate gyrus; Or, oriens layer of the hippocampus; Py, pyramidal cell layer of the hippocampus; Rad, stratum radiatum of the hippocampus.

The depth profile of the evoked fEPSPs was recorded along a vertical tract starting from the granule cell layer of the DG and decreasing the depth, stepwise, with 5 min intervals up to the CA1 cell layer. This strategy (of beginning in the DG) was followed so that we could obtain a proper orientation as to the location of the electrode as we moved toward the CA1 region. The recordings were taken from a rat under anesthesia (Figure 2).

The stimulation of the medial perforant path typically evoked a large positive-going field response with a superimposed population spike at the DG granule cell layer. Whereas the responses evoked at the CA1-slm are much smaller in size and usually comprise volume-conducted currents as well as long latency negative going fEPSP.

Stimulation of the Schaffer collaterals *in vitro* evokes a negative-going fEPSP at the CA1-sr and positive one at the CA1-slm. Conversely the stimulation of the perforant path evokes a positive-going fEPSP at the CA1-sr and negative one at the CA1-slm (Colbert and Levy, 1992; Dvorak-Carbone and Schuman, 1999a; Remondes and Schuman, 2003; Speed and Dobrunz, 2009). To visualize if the same profile could be evoked in the intact brain, an additional stimulation electrode was placed in the Schaffer collaterals and independent recordings were obtained for each stimulation site from a single recording electrode placed at CA1-slm for different distances from bregma (Figure 2).

### Characteristics of pp-CA1 potentials: comparison with Sc-CA1 potentials

Typically, stimulation of the Schaffer collateral fibers resulted in a single negative going potential in the CA1-sr. Whereas, the stimulation of the perforant path elicited a composite potential with a complex shape.

**Figure 3:**
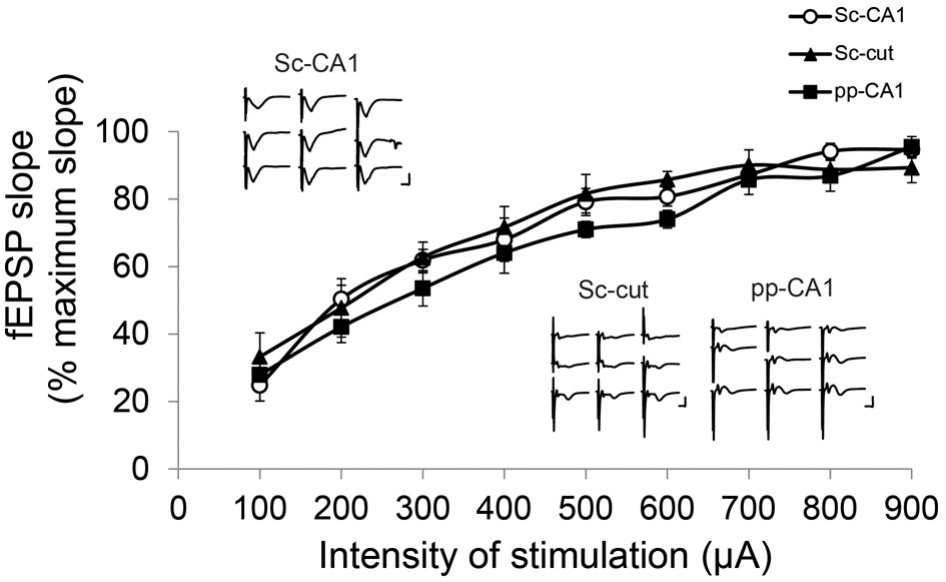
**Input/Output characteristics for the Sc-CA1, pp-CA1, and Sc-cut synapse.** The fEPSP slope was recorded as the maximum slope from the beginning of the potential to the first minimum value for each subject. For each intensity of stimulation the values are expressed as percentage of the highest value obtained. There was no significant difference between Sc-CA1, SC-severed (Sc-cut) or pp-CA1 groups (*n* = 8 for Sc-CA1, *n* = 8 for Sc-cut and *n* = 9 for pp-CA1). Typical evoked responses from Sc-CA1, Sc-cut, and pp-CA1 animals embedded in the chart. Increasing the stimulation intensity stepwise (100–900 μA, step size 100 μA) complicated the analysis of the fEPSPs from the pp-CA1 and Sc-cut synapse to same extent. Vertical scale bar: 3 mV, horizontal scale bar: 5 ms.

Increasing the stimulation intensity in a stepwise manner did not affect the shape of the Sc-CA1 potentials drastically, but resulted in a steeper slope and increased amplitude. However, the same procedure increased the complexity of the pp-CA1 potentials by adding volume-conducted currents due to the stimulation of the DG, and a second negative-going potential, due to the subsequent activation of the CA3 region (Figure 3). Severance of the Sc fibers neither changed the general appearance nor the response to increase in stimulation for the pp synapse. For all groups the I/O curves had similar characteristics (Sc-CA1 compared to pp-CA1 or Sc-cut for all stimulation intensities *p* > 0.05; Figure 3). Consequently the stimulation intensity used for plasticity experiments corresponded to the range of 100–250 μA (intensity of stimulation to elicit 40% of the maximum fEPSP slope) for all groups.

### Paired pulse responses are different at pp-CA1 and Sc-CA1 synapses

To access more information about the electrophysiological characteristics of the intact pp-CA1 synapse in freely behaving animals, ultra short-term plasticity was examined by the means of paired-pulse ratios for stimuli with different interpulse durations.

Stimulation of a synapse with two pulses with a very short interpulse interval (typically ranging in milliseconds) results in ultra short-term plasticity that is thought to reflect mainly, but not exclusively, the presynaptic properties of the synapse (Thomson, 2000; Zucker and Regehr, 2002). Previously, it has been shown *in vitro* that the paired-pulse response of the Sc-CA1 vs. the pp-CA1 is different for juvenile rats but not young adults (Speed and Dobrunz, 2009). Whether the same relationship is present for freely behaving rats was not previously investigated.

**Figure 4:**
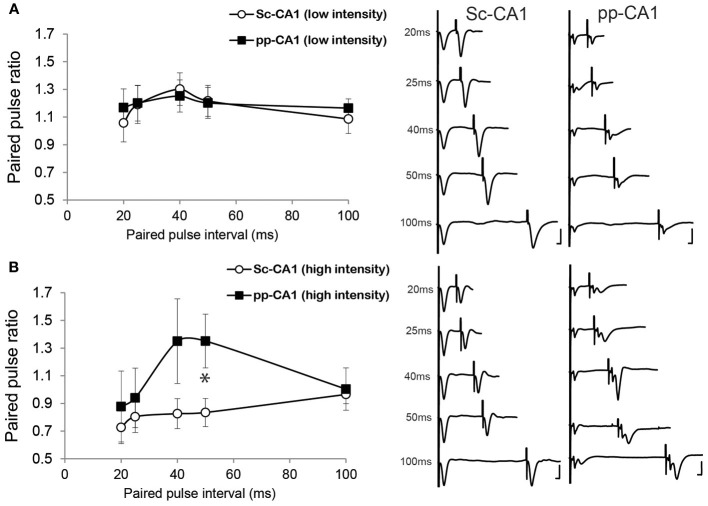
**The paired-pulse ratio is different for pp-CA1 and Sc-CA1 synapse depending on stimulus intensity. (A)** Left: Paired-pulse ratio profiles for pp-CA1 and Sc-CA1 synapses at low-intensity stimulation (corresponding to the intensity necessary to evoke 40% of the maximum). Right: Example of fEPSPs evoked from corresponding synapse and interstimulus interval (Vertical scale bar: 4 mV, horizontal scale bar: 5 ms for all) **(B)** Left: Paired-pulse ratio profiles for pp-CA1 synapses and for the Sc-CA1 synapses at high-intensity levels (double the low-intensity or maximum of 400 μA). Increasing the stimulation intensity did not result in a significant change for the pp-CA1 synapse but led to paired-pulse depression at all interstimulus intervals for the Sc-CA1 synapse. The paired-pulse intervals used were: 20, 25, 40, 50, and 100 ms for both groups. All the recordings were collected from awake unrestrained but stationary animals. ^*^indicates *p* < 0.05 (Student's *t*-test). Right: Example of fEPSPs evoked from corresponding synapse and interstimulus interval (Vertical scale bar: 4 mV, horizontal scale bar: 5 ms).

Low- intensity stimulation revealed a similar profile of the paired pulse ratio for both the pp-CA1 and the Sc-CA1 synapse (Figure 4A). At high-intensity stimulation, 50 ms inter-pulse intervals resulted in a significant difference between the Sc-CA1 and pp-CA1 animals (*p* < 0.05; *n* = 7 for Sc-CA1, *n* = 6 for pp-CA1) (Figure 4B). Increasing the intensity of stimulation drastically changed the paired-pulse ratio profile for the Sc-CA1 synapse for all inter-pulse intervals (*p* < 0.05; *n* = 7) but not for the pp-CA1 synapse (no significant difference at different intensities for all inter-pulse intervals, *p* > 0.05). High-intensity stimulation complicated the evoked responses from pp-CA1 synapse and resulted in a high variability within the group.

### Long-term potentiation is different at pp-CA1 and Sc-CA1 synapses

Previously it was shown *in vitro* that LTP is induced at the pp-CA1 synapse by application of HFS (Remondes and Schuman, 2002). A typical protocol that elicits LTP at the Sc-CA1 synapse *in vivo* consists of single or multiple 100 Hz burst(s) of stimulation of afferent fibers (Kemp and Manahan-Vaughan, 2004). Although burst(s) of 100 Hz result in robust LTP at the Sc-CA1 synapse, it evokes short-term potentiation (STP) at perforant path-dentate gyrus synapses (Kulla and Manahan-Vaughan, 2000). In contrast, 200 Hz tetanic stimulation reliably induces persistent LTP in the DG, as previously shown in our laboratory (Kulla and Manahan-Vaughan, 2000, 2008; Manahan-Vaughan and Kulla, 2003).

**Figure 5:**
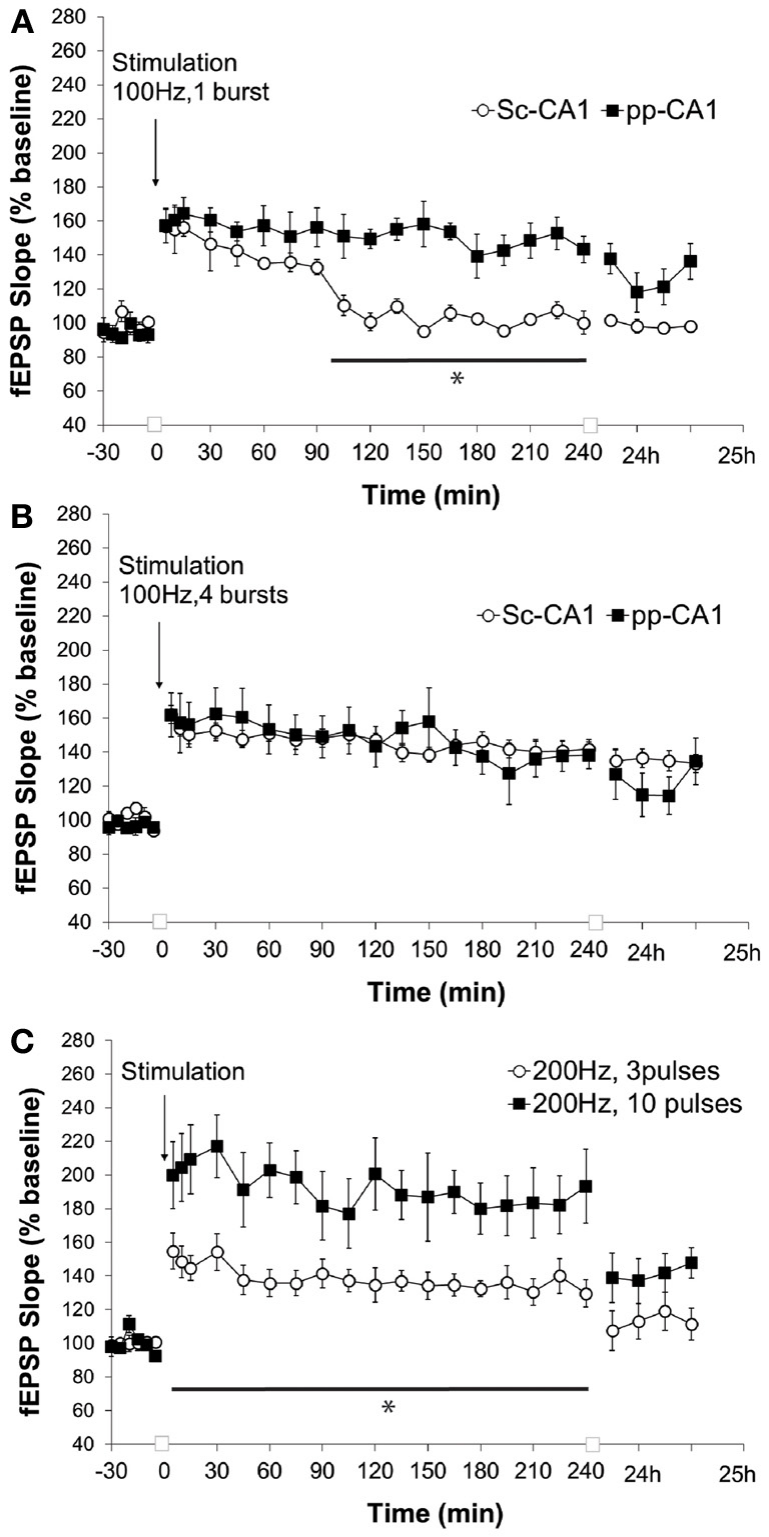
**High frequency stimulation (100 Hz) at different strengths elicits LTP of differing durations at the pp-CA1 and Sc-CA1 synapses. (A)** High frequency stimulation (HFS) with a weak tetanus of 100 Hz (1 burst of 100 pulses) resulted in short term potentiation at the Sc-CA1 synapse and LTP at the pp-CA1 synapse in freely behaving rats. Synaptic potentiation at pp-CA1 synapses was significantly more prolonged than potentiation at SC-CA1 synapses (ANOVA). The line with the asterisk indicates individual time-points that were determined to be significant in the pp-CA1 responses compared to the Sc-CA1 responses, using *post-hoc* Fisher's LSD test. **(B)** Strong HFS with 100 Hz (4 bursts of 100 pulses) elicited comparable potentiation at the two synapses. **(C)** HFS with a weak or strong tetanus of 200 Hz [3 bursts (wHFS) or 10 bursts (HFS)] resulted in LTP of differing amplitude at pp-CA1 fEPSPs from freely behaving rats. LTP elicited with 200 Hz, 10 pulses was significantly larger than LTP elicited with 200 Hz, 3 pulses (ANOVA). The line with the asterisk indicates individual time-points in the 10 pulse group that were determined to be significant from the 3 pulse group, using *post-hoc* Fisher's LSD test.

High-frequency stimulation of the perforant path to CA1 synapse with a single burst of 100 pulses at 100 Hz (0.1 ms stimulus duration) resulted in synaptic potentiation that lasted for over 4 h whereas for Sc-CA1 the same stimulation elicited potentiation that lasted ca. 90 min (*p* < 0.05). The strength of potentiation at the two synapses was significantly different [ANOVA, *F*_(1, 246)_ = 135.72; *p* < 0.001, *n* = 6 for Sc-CA1 and *n* = 7 for pp-CA1, Figure 5A]. Increasing the strength of stimulation to four bursts of 100 pulses at 100 Hz induced comparable potentiation at both pp-CA1 and Sc-CA1 synapses [ANOVA, *F*_(1, 275)_ = 0.19; *p* > 0.05, *n* = 6 for Sc-CA1 and *n* = 8 for pp-CA1; Figure 5B].

Application of a “weak” or “strong” tetanus of 200 Hz (i.e., 3 or 10 bursts of 15 stimuli, respectively) resulted in robust early potentiation with differing magnitudes at the pp-CA1 synapse (Figure 5C). The difference in the degree of potentiation remained for the following 4 h, but by 24 h post-tetanus no difference in the level of potentiation was evident. The magnitude of potentiation induced at the pp-CA1 synapse via weak and strong 200 Hz HFS was significantly different [ANOVA, *F*_(1, 230)_ = 127.19; *p* < 0.001; *n* = 6, Figure 5C]. Tetanisation at 200 Hz led to epileptifom seizures at the Sc-CA1 synapse that was typically followed by a loss of the fEPSP (not shown). Thus, this stimulation could not be used to elicit LTP at the Sc-CA1 synapse.

### Long-term depression is different at pp-CA1 and Sc-CA1 synapses

**Figure 6:**
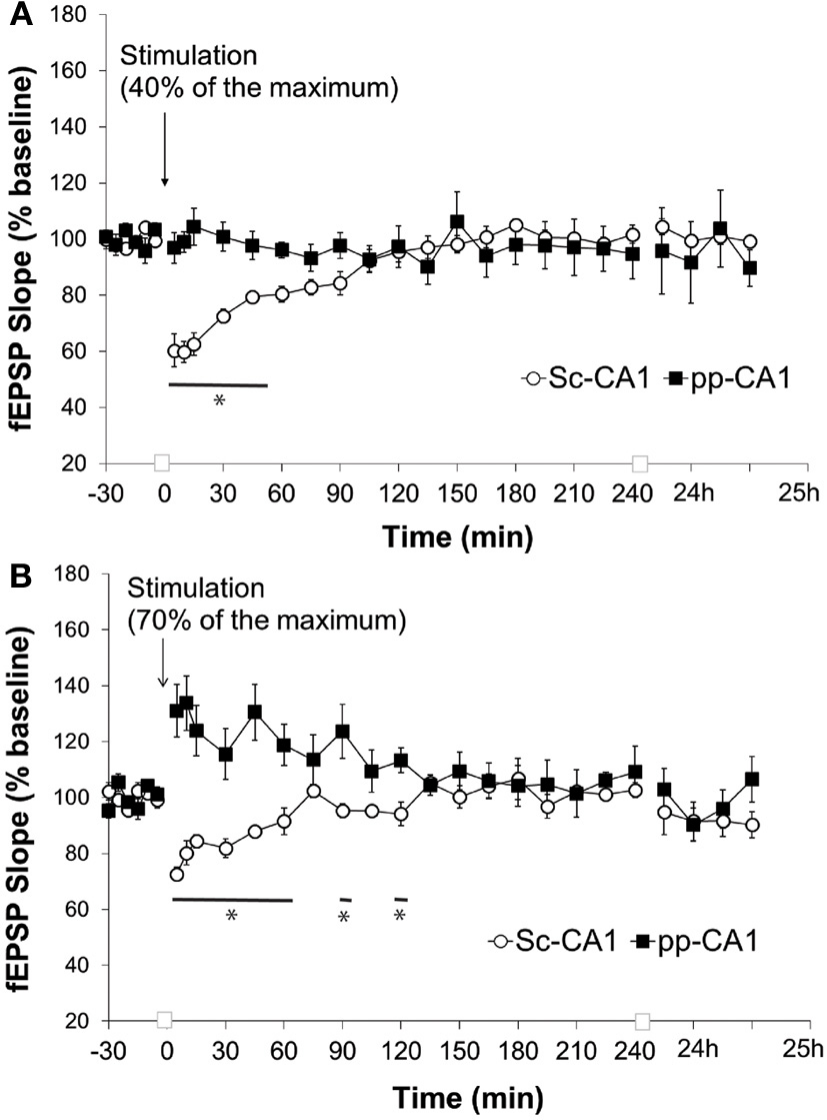
**Low frequency stimulation induces short-term depression at the Sc-CA1 synapse irrespective of stimulation intensity but potentiates the pp-CA1 synapse with high-intensity. (A)** Low frequency stimulation (LFS, 1 Hz, 600 pulses) with low-intensity stimulation (to evoke 40% of the maximum evoked response) did not have any effect at the pp-CA1 synapse in freely behaving rats, whereas short-term depression was elicited at SC-CA1 synapses. Sc-CA1 depression was significant compared to evoked potentials at pp_CA1 synapses (ANOVA). The line with the asterisk indicates individual time-points in SC-CA1 responses that were determined to be significant from the pp-CA1 responses, using *post-hoc* Fisher's LSD test. **(B)** LFS (1 Hz, 600 pulses) with high-intensity stimulation (to evoke 70% of the maximum evoked response) resulted in potentiation at the pp-CA1 synapse in freely behaving rats and short-term depression in the Sc-CA1 synapses. The lines marked with asterisks indicate individual time-points in SC-CA1 responses that were determined to be significant from the pp-CA1 responses, using *post-hoc* Fisher's LSD test.

Low frequency stimulation (LFS) of the pp-CA1 synapse at 1 Hz at a low-stimulus intensity (40% of the maximum obtained slope from the I/O curve) for 10 min did not reveal any difference at the pp-CA1 evoked responses with respect to baseline measurements. The average of the slope of the potentials was calculated as 97 ± 5% (mean percent of baseline) at 5 min after the stimulation and did not change significantly. In contrast, the same stimulation elicited short-term depression at Sc-CA1 synapses that was statistically significant from the pp-CA1 response [ANOVA, *F*_(1, 226)_ = 11.80; *p* < 0.001; *n* = 6 for Sc-CA1 and pp-CA1, Figure 6A]. Increasing the stimulation intensity (to evoke 70% of the maximum evoked response seen in the I/O relationship), during the application of the LFS, has been shown in other studies to induce either STD or LTD at Sc-CA1 synapses (Kemp and Manahan-Vaughan, 2004). Unexpectedly, in our study increasing the stimulation intensity potentiated the evoked responses at the pp-CA1 synapse (mean percent of baseline for LFS at 5 min was 131 ± 9%) that remained about an hour (mean percent of baseline for LFS at 60 min; 119 ± 7). The response to the stimulation was significantly different for pp-CA1 and Sc-CA1 synapses [ANOVA, *F*_(1, 249)_ = 74.60; *p* < 0.001; *n* = 6 for Sc-CA1 and *n* = 7 for pp-CA1, Figure 6B].

### Synaptic plasticity occurs in the CA1 region under conditions where the schaffer collateral-CA1 input is not intact

**Figure 7:**
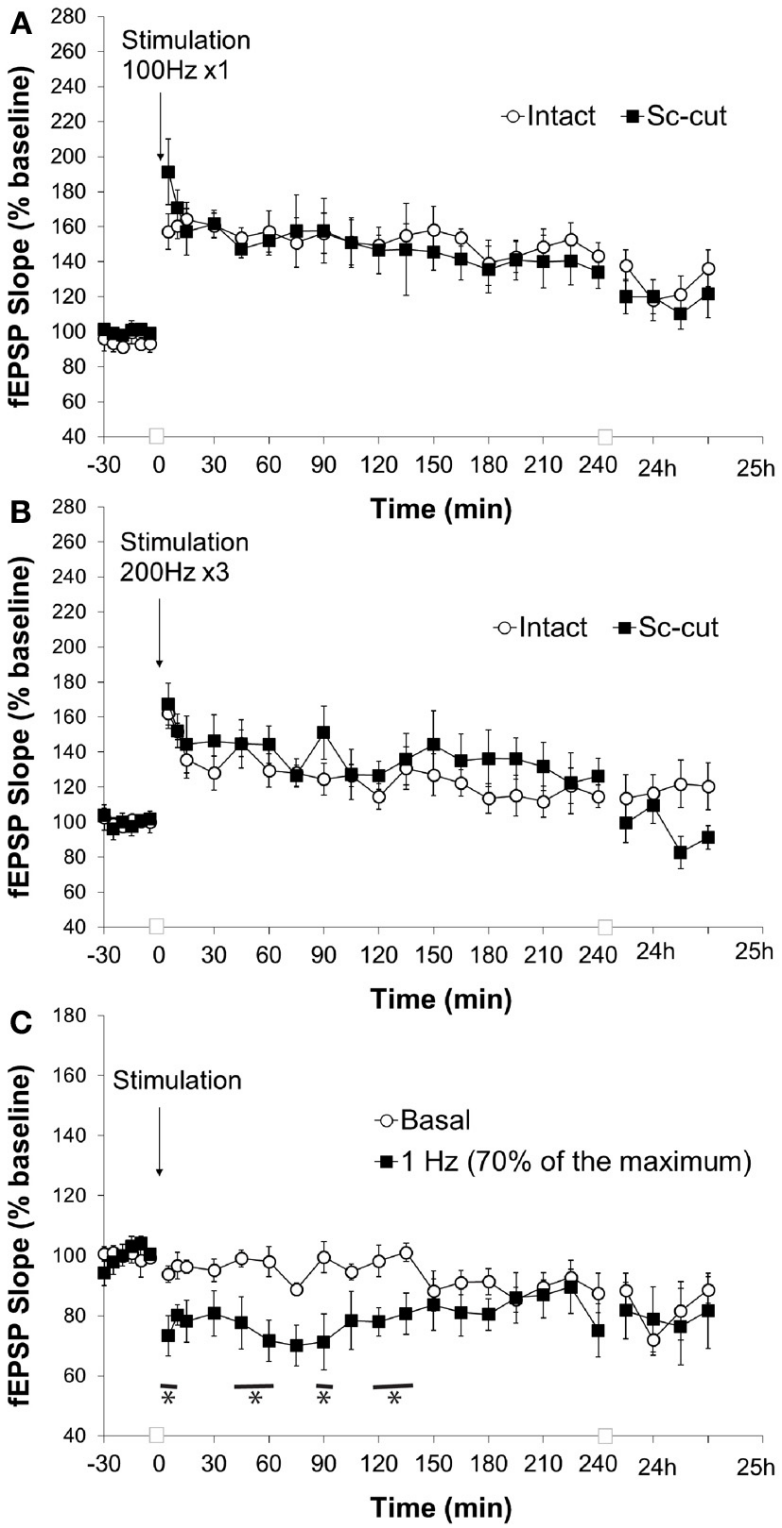
**Severence of CA3 input affects the response of the pp-CA1 synapse to LFS but not HFS. (A)** High frequency stimulation (HFS) with a weak tetanus of 100 Hz (1 burst of 100 pulses) resulted in potentiation in freely behaving rats with intact and Sc-cut conditions. **(B)** High frequency stimulation with a weak tetanus of 200 Hz (3 burst of 15 pulses) resulted in potentiation in freely behaving rats with intact and Sc-cut conditions. **(C)** Low frequency stimulation (1 Hz, 600 pulses) resulted in depression at the pp-CA1 synapse in freely behaving rats with a severed Schaffer collateral input compared to responses evoked in the same synapses by test-pulse stimulation (ANOVA). The lines marked with asterisks indicate individual time-points representing evoked responses after 1 Hz stimulation that were determined to be significant from test-pulse evoked responses, using *post-hoc* Fisher's LSD test.

We cannot exclude that the absence of LTD at the pp-CA1 synapses resulted from an additional influence of the Sc-CA1 synapses on evoked responses. Therefore, we severed the CA3 sub-region via chronic implantation of a stainless steel plate to isolate the pp-CA1 synapse. Evoked responses revealed a larger pp-CA1 component and a smaller Sc-CA1 component compared to animals whose Schaffer collaterals were intact (Figure 7A). This suggests that a significant portion of Schaffer collateral inputs to the CA1 synaptic population, from which we recorded, were severed.

Stimulation with weak HFS (100 Hz) resulted in a comparable potentiation at the pp-CA1 synapse in the intact hippocampus (Figure 5) and where the connection to the CA3 region was severed (Figure 7A) (mean percent of baseline for the intact vs. Sc-cut animals at 5 min; 157 ± 10% vs. 191 ± 19%). After 4 h, a potentiation of smaller amplitude was still present in both groups (mean percent of baseline for intact vs. Sc-cut animals at 240 min; 143 ± 8% vs. 134 ± 9%). A similar pattern was observed after 24 h (mean percent of baseline for intact vs. Sc-cut animals at 25 h; 136 ± 10% vs. 122 ± 14%). Further statistical analysis revealed no significant difference between the groups for the responses to the stimulation [ANOVA, *F*_(1, 246)_ = 1.46; *p* > 0.05; *n* = 7 for intact and *n* = 6 for Sc-cut; Figure 7A].

Similar to high-frequency stimulation with 100 Hz (1 burst, 100 pulses), stimulation at 200 Hz (3 bursts, 15 pulses) did not reveal any difference in responses for the intact and Sc-severed animals. The mean percent of baseline at 5 min after the stimulation increased to 162 ± 9% vs. 167 ± 12% for the intact and Sc-cut group, respectively. After 4 h, the potentiation was still present in both groups (mean percent of baseline for intact vs. Sc-cut animals at 240 min; 115 ± 6% vs. 126 ± 10%). Statistical analysis revealed no significant difference in the responses to the stimulation with 200 Hz [ANOVA, *F*_(1, 319)_ = 0.28; *p* > 0.05; *n* = 8; Figure 7B].

The low-frequency stimulation protocol that elicited changes in the evoked synaptic responses at the pp-CA1 synapse in the intact brain was utilized for the Sc-cut animals to study the possible effect of the Sc input on the plasticity of the pp-CA1 synapse. Interestingly, under these conditions, 1 Hz (600 pulses) stimulation (70% intensity) resulted in synaptic depression that was 73 ± 6% of pre-LFS levels, 5 min after conclusion of LFS (compared to 94 ± 2% in test-pulse stimulated controls). Responses recovered to baseline levels after ca. 2 h. The response to the stimulation was significantly different from the test-pulse control [ANOVA, *F*_(1, 221)_ = 40.88; *p* < 0.001; *n* = 6, Figure 7C].

## Discussion

This study describes the first attempt to characterize synaptic plasticity at the perforant path(pp)-CA1 synapses in awake animals. First, electrophysiological procedures were established to study the direct cortical input from the EC to the hippocampal CA1 sub-region in freely behaving adult rats. Furthermore, the two synapses at the CA1 sub-region responded differently to paired-pulse stimulation, depending on the intensity of stimulation. High-frequency stimulation (HFS) elicited LTP at the pp-CA1 synapse, the amplitude and duration of which depended on the frequency and strength of the protocol used. On the other hand, application of typical low-frequency stimulation (LFS) protocol that elicits synaptic depression at the Sc-CA1 synapse, either failed to induce plastic changes at the pp-CA1 synapse or resulted in potentiation of the evoked responses, depending on the intensity of stimulation. Severance of the input from the CA3 sub-region had no impact on the profile of LTP elicited by HFS, but enabled LTD following LFS, suggesting a modulatory role of the tri-synaptic input on the plasticity of the pp-CA1 synapse.

It has been well-documented that cutting the CA3 region and dissociating the DG leaves an isolated CA1 region that enables the electrophysiological characterization of the pp-CA1 synapse *in vitro* (Empson and Heinemann, 1995; Remondes and Schuman, 2003; Speed and Dobrunz, 2009). Previous reports on the characterization of the direct cortical innervation to the hippocampal CA1 in anesthetized rats, pointed to the volume conduction from the strongly activated DG (and possibly the CA3) as a direct concomitant of the monosynaptically-recorded EPSPs from the CA1 (Stringer and Colbert, 1994; Leung, 1995). Similarly, we found that increasing the stimulation intensity complicates the pp-CA1 response in awake rats, possibly due to the larger number of perforant path fibers recruited not only for the CA1 region but also for the DG and CA3 regions. Irrespective of the signal contamination, the pp-CA1 synapse displayed differential pre- and post-synaptic responses compared to the Sc-CA1 and pp-DG synapses in awake rats.

The degree of paired-pulse facilitation or depression relates to the probability of transmitter release, the number of release sites as well as the quantal amplitude (Thomson, 2000; Zucker and Regehr, 2002). Our observation that the paired-pulse responses of the Sc-CA1 and pp-CA1 synapses are similar, when low stimulus intensity is used, is in line with the previous reports from *in vitro* preparations (Speed and Dobrunz, 2009). If we used a higher stimulus intensity, paired-pulse depression was favored at the Sc-CA1 synapse, similar to effects reported in awake mice (Madronal et al., 2009). Paired-pulse depression was not evident at pp-CA1 synapses, however. This is consistent with the fact that pp-CA1 terminals have a lower release probability compared to the Schaffer collaterals (Ahmed and Siegelbaum, 2009) and they are less prone to synaptic vesicle depletion. When stimulated with high-intensity the interpulse duration of 50 ms let to different paired-pulse response for the two synapses that is in line with a previous report identifying distinct properties of NMDA receptor mediated current for pp-CA1 synapse compared to Sc-CA1 synapse (Otmakhova et al., 2002). It cannot be completely excluded that high intensity stimulation might contaminate the pp-CA1 response through adding a DG component. We observed, however, that the paired-pulse ratio for the inter-pulse intervals used in this study was strongly depressed for DG responses, when the stimulation intensity was high enough to evoke a population spike in the DG in the first fEPSP of the pair. This suggests that the second evoked fEPSP in the pp-CA1 response is not likely to contain a significant contribution from the DG.

Induction of LTP at the pp-CA1 synapse has had a contradictory outcome *in vitro* (Colbert and Levy, 1992; Remondes and Schuman, 2002, 2003). We consistently observed LTP (>24 h) following HFS that did not return to basal levels even 24 h after weak (200 Hz) HFS, suggesting that the pp-CA1 synapse is capable of expressing robust and very persistent LTP that is distinct from Sc-CA1 LTP. LTP induced with 100 Hz was significantly different in duration but not in magnitude in pp-CA1 and Sc-CA1 synapses. However, with stronger stimulation both synapses displayed comparable LTP.

LFS of the perforant path (1 Hz for 10 or 15 min) results in homosynaptic LTD of pp-CA1 synapses *in vitro* (Dvorak-Carbone and Schuman, 1999a; Wöhrl et al., 2007). Dvorak-Carbone and Schuman, in their *in vitro* study (1999a), showed that LTD at the pp-CA1 synapse can be induced even with a protocol that is not strong enough to affect the Sc-CA1 synapse. In the present work, however, LFS that induces short-term depression (STD) at the Sc-CA1 synapse in freely behaving animals (Manahan-Vaughan, 2000) failed to induce any change in the basal synaptic transmission at the pp-CA1 synapse under similar conditions.

One of the effective factors in induction of long-term plasticity is stimulation intensity (Leung and Au, 1994). Thus, stimulation intensity during LFS was increased to evoke responses that reflect fEPSPs that comprise 70% of the maximum obtained in the input-output curve. LFS given at this intensity evokes persistent LTD in Sc-CA1 synapses (Manahan-Vaughan and Braunewell, 1999, 2005; Kemp and Manahan-Vaughan, 2004) and in mossy fiber- CA3 synapses(Hagena and Manahan-Vaughan, 2010). Interestingly, increasing the stimulation intensity during LFS resulted in synaptic potentiation (<3 h) at the pp-CA1 synapse *in vivo*. The simplest explanation for this surprising outcome is interference derived from the increased excitability and lack of flexibility of the pp-CA1 synapse, in relationship to the increased stimulation intensity. A positive-going inflection appeared in the fEPSP after the LFS was given- thus complicating the overall picture, as it suggests that volume-conduction from the activated DG contaminated the fEPSP (Stringer and Colbert, 1994; Buzsaki et al., 1995; Leung et al., 1995). Nonetheless, possible indirect effects of volume conduction from the Sc-CA1 and pp-DG activation do not explain the potentiation in its entirety since both the Sc-CA1 and pp-DG synapses undergo LTD following LFS at 1 Hz (and even at a similar stimulation intensity as used here) (Manahan-Vaughan and Braunewell, 1999, 2005; Kemp and Manahan-Vaughan, 2004).

Evidence exists, however, that gradually evolving LTP can be induced at the Sc-CA1 synapse if the LFS (1 Hz) is applied for short periods (3-5 min) (Lante et al., 2006a,b). Furthermore, another study suggests distance dependent differential effects of LFS on the CA1 apical dendrites that span the CA1-sr layer (Parvez et al., 2010). What might be the functional significance of LFS-induced potentiation at the pp-CA1 synapse and does it actually occur under physiological conditions? Indeed, slow oscillations with a frequency of around 1 Hz were implicated as a state in slow-wave-sleep that shows a current sink in the CA1-slm layer (Wolansky et al., 2006). Interestingly, the response of the pp-CA1 synapse during the spontaneously occurring slow oscillations appears amplified whereas the pp-DG synapse has a reduced response (Schall and Dickson, 2010). Thus, it is quite possible that the consolidation of memories during sleep is reinforced by slow oscillations in different hippocampal sub-regions, as proposed previously (Brun et al., 2002; Remondes and Schuman, 2004).

Whether the role of the direct input on the CA1 is independent or only modulatory to the trisynaptic circuit was also addressed in our study. *In vitro* experiments suggest a potent modulatory role for the perforant path input, exerted on synaptic transmission and synaptic plasticity of the Sc-CA1 synapse (Levy et al., 1998; Dvorak-Carbone and Schuman, 1999b; Remondes and Schuman, 2002; Judge and Hasselmo, 2004; Ang, 2005; Dudman et al., 2007; Wöhrl et al., 2007; Izumi and Zorumski, 2008). LFS of the perforant path results in either transient depression (<5 min) (Izumi and Zorumski, 2008) or no effect (Dvorak-Carbone and Schuman, 1999a) on the Schaffer collateral evoked responses. Under basal conditions the effect of Sc input on pp-CA1 fEPSPs was not detectable with our methodology, which may be due to spared fibers. Previously Scharfman et al. (1998) observed no drastic change in fEPSPs from pp-CA1 synapse after selective chemical lesion of EC Layer III that suggests even partial integrity of the synapse is sufficient to evoke fEPSP. Whereas the more demanding conditions reveal the functional contribution of the Sc.

We observed that animals with severed CA3 did not differ significantly from the intact group when HFS protocols were applied, suggesting no interference of the Schaffer collateral input to the LTP induction at the pp-CA1 synapse. Interestingly, LFS (1 Hz, 10 min; 70%) that potentiated the pp-CA1 synapse in intact animals resulted in LTD when the CA3 was severed. The induction of LTD is not attributable to non-specific brain damage since induction of LTD is an active process that requires anatomical as well as molecular integrity of the synapse (Mulkey and Malenka, 1992; Bear and Abraham, 1996; Hrabetova and Sacktor, 1997).

The successful induction of LTD that we observed when the Schaffer collateral input was severed, is well in line with *in vitro* findings where the CA3 is separated from CA1 for technical reasons (Dvorak-Carbone and Schuman, 1999a). These results also favor the possibility that the Sc-CA1 synapse is actively responding to the outcome of LFS at the pp-CA1 synapse. This may possibly occur through the activation of the feed-back inhibitory system (Maccaferri and Mcbain, 1995). Another conclusion is that induction of LTP at the pp-CA1 synapse with different HFS frequencies is not affected by the Schaffer collateral input, suggesting differential network activity of the hippocampal sub-regions to LTP and LTD protocols. Effects are not likely to have been influenced by gliosis in the area where the severance of afferents was implemented: Dinocourt et al. (2011) reported that gliosis and damage resulting from *in vivo* transection of the Schaffer collaterals is localized to the region of severance. However, the same authors reported an increased excitability in the CA1 *in vitro* as a result of Schaffer collateral transection. We neither found any change in the general profile of the fEPSP, nor the input-output responses, nor in the response to HFS. This suggests that the LTD we successfully induced after Schaffer collateral severance does not derive from excitability changes *per se*.

The difference in synaptic plasticity responses at the pp-CA1 and Sc-CA1 synapses may well be a result of the modulatory neurotransmission that differentially affects the Sc-CA1 and the pp-CA1 synapses (Otmakhova and Lisman, 2000). Compared to the Sc-CA1 synapse we did not observe a gradually decaying LTP in the pp-CA1 synapse, for example. That might be a result of disinhibition by dopamine (Ito and Schuman, 2007) leading to a ceiling effect with application of single pulse HFS (100 Hz). Changing the stimulation frequency and consequently the temporal pattern of stimulation might change the extent and direction of modulatory effect.

Several theories have postulated a special role of the pp-CA1 synapses in hippocampal information storage (Lisman and Otmakhova, 2001; Treves, 2004; Hasselmo and Eichenbaum, 2005; Rolls and Kesner, 2006). Our data support that synaptic plasticity at this synapse is distinct from the main synapses of the trisynaptic network: the thresholds for LTP induction are lower and LTP evoked at lower frequencies is more robust than LTP evoked at the Sc-CA1 synapse, (this study) or in pp-dentate gyrus, mossy fiber-CA3 or commissural associational synapses of the CA3 region in freely behaving rats (Manahan-Vaughan et al., 1998; Kemp and Manahan-Vaughan, 2008; Hagena and Manahan-Vaughan, 2012). LTD is induced at the isolated pp-CA1 synapses but appears to be subjected to a strong modulatory control by Sc-CA1 synapses under conditions where all hippocampal synapses are intact. The suppression of LTD by Sc-CA1 synapses may serve to amplify or support LTP that is elicited at the pp-CA1 synapse and thus specifically enable this form of synaptic plasticity within this sparse synaptic system. Whether this relates to the provision of efference copy to the CA1 region (Lisman and Otmakhova, 2001), supports temporal encoding (Treves, 2004; Hasselmo and Eichenbaum, 2005; Rolls and Kesner, 2006) or error detection (Izumi and Zorumski, 2008) remains to be clarified.

### Conflict of interest statement

The authors declare that the research was conducted in the absence of any commercial or financial relationships that could be construed as a potential conflict of interest.
